# Amount and timing of physical activity in relation to sleep quality in the general middle-aged Dutch population: A cross-sectional analysis

**DOI:** 10.1016/j.pmedr.2025.103035

**Published:** 2025-03-18

**Authors:** Charlotte Andriessen, Femke Rutters, Joris Hoeks, Andries Kalsbeek, Raymond Noordam, Frits R. Rosendaal, Diana van Heemst, Jean-Pierre Després, Parminder Raina, David J.T. Campbell, Patrick Schrauwen, Renée de Mutsert, Jeroen H.P.M. van der Velde

**Affiliations:** a Department of Nutrition and Movement Sciences, Maastricht University Medical Center, Maastricht, the Netherlands; b Department of Epidemiology and Data Science, Amsterdam UMC, location VUmc, Amsterdam, the Netherlands; c Amsterdam Public Health, Health Behaviors & Chronic Diseases, Amsterdam UMC, Amsterdam, the Netherlands; d Department of Endocrinology and Metabolism, Amsterdam UMC, University of Amsterdam, AZ, Amsterdam, the Netherlands; e Hypothalamic Integration Mechanisms, Netherlands Institute for Neuroscience (NIN), An Institute of the Royal Netherlands Academy of Arts and Sciences (KNAW), BA, Amsterdam, the Netherlands; f Department of Internal Medicine, Section of Gerontology and Geriatrics, Leiden University Medical Center, Leiden, the Netherlands; g Department of Clinical Epidemiology, Leiden University Medical Center, Leiden, the Netherlands; h Centre de recherche sur les soins et les services de première ligne, Université Laval, Québec City, Québec, Canada; i Department of Health Research, Evaluation and Impact, Faculty of Health Sciences, McMaster University & McMaster Institute for Research on Aging, Hamilton, Ontario, Canada; j Department of Medicine, Cumming School of Medicine, University of Calgary, Calgary, Canada; k Department of Community Health Sciences, Cumming School of Medicine, University of Calgary, Calgary, Canada; l Department of Cardiac Sciences, Cumming School of Medicine, University of Calgary, Calgary, Canada

**Keywords:** Epidemiology, Physical activity, Sleep, Zeitgeber, Circadian rhythm, Exercise

## Abstract

**Objectives:**

To examine whether the amount and timing of moderate-to-vigorous physical activity (MVPA) was associated with sleep quality and duration in the general population.

**Methods:**

This is a cross-sectional analysis of data of a Dutch cohort collected between 2008 and 2012. Timing of physical activity (measured using an accelerometer) was categorized as performing most MVPA in morning (06:00–12:00), afternoon (12:00–18:00), evening (18:00–00:00), or even distribution of MVPA over the day (reference). Sleep quality was assessed using the Pittsburgh Sleep Quality Index (PSQI). We estimated OR with 95 % CI of a poor score on individual PSQI components and global PSQI score using logistic regression while adjusting for relevant covariates.

**Results:**

We analyzed 736 participants, of whom 57 % women, aged 56 (6) years, BMI 26.1 (4.2) kg/m^2^). Amount of MVPA (hours/day) was associated with lower odds of fatigue-related dysfunction during daytime (OR: 0.54 0.32–0.94), but not with global PSQI score. Participants who performed most MVPA in the morning were less likely to report sleep disturbances (OR: 0.23, 95 % CI: 0.09–0.60), compared to participants with an even distribution of. Timing of MVPA was not associated with global PSQI score nor other components and CI were large.

**Conclusions:**

Differences in sleep quality are unlikely to be biological mechanisms underlying the previously shown associations between timing of physical activity and metabolic health.

## Highlights

• Moderate-vigorous physical activity is associated with daytime functioning.
• Mainly, no associations of timing of moderate-vigorous activity with sleep quality.
• Morning moderate-vigorous activity only was associated with fewer sleep disturbances.
• As confidence intervals of our findings were wide, larger studies are needed.

## 1 Introduction

About one third of the Western population reports dissatisfaction with their sleep, which is often described as too short, too light or generally unsatisfying (Ohayon and Reynolds III, 2009). Poor sleep has been associated with a plethora of medical conditions including, obesity and type 2 diabetes (Bacaro et al., 2020; Javaheri et al., 2011; Rutters et al., 2016; Xi et al., 2014; Cappuccio et al., 2010). Physical activity has been proposed as an effective health behavior modification to ameliorate sleep problems (Kredlow et al., 2015). The mechanisms underlying the beneficial effect of physical activity on sleep are unclear, but may involve a reduction in depression (O'Neal et al., 2000) and anxiety (O'Connor et al., 2000) symptoms, a temperature elevation before bedtime (Horne and L. Staff, 1983), and alleviating obstructive sleep apnea (Van Offenwert et al., 2019).

Most evidence linking the amount of physical activity and sleep originates from human intervention studies. For instance, a meta-analysis including 66 controlled trials in healthy adults showed that an acute bout of physical activity positively affects sleep quantity, whereas regular physical activity positively affects both sleep duration and quality, obtained either via self-report or with biological measurements (e.g. polysomnography and electroencephalography) (Kredlow et al., 2015). Only few observational studies investigated the association between habitual physical activity and sleep characteristics (Gubelmann et al., 2018; Kline et al., 2013; Lang et al., 2013; McClain et al., 2014; Spörndly-Nees et al., 2017), and mostly they relied on self-reported physical activity and sleep (Kline et al., 2013; Lang et al., 2013; Spörndly-Nees et al., 2017). The role of moderate-to-vigorous physical activity (MVPA) has not been thoroughly investigated in relation to sleep in the general population., while this may be relevant for sleep, as MVPA results in greater physiological adaptations than low-intensity physical activity.

Furthermore, physical activity is also an important “Zeitgeber” for the internal timing system regulating sleep- and wake rhythm and disruptions in this circadian rhythm has been linked to metabolic disturbances (Stenvers et al., 2019). Recent studies have shown that performing regular or high-intensity exercise during late daytime resulted in reduced 24-h glucose levels and improved insulin sensitivity compared with exercise in the morning in men with impaired glucose metabolism or diabetes (Mancilla et al., 2021; Savikj et al., 2019). Likewise, we recently observed in a general middle-aged population that individuals performing most MVPA in the afternoon and evening, but not in the morning had lower insulin resistance, compared with those with an even distribution of MVPA over the day (van der Velde et al., 2022). It is yet unknown what potential mechanisms underly this association. Because sleep quality is important for metabolic health (Bacaro et al., 2020; Javaheri et al., 2011; Rutters et al., 2016; Xi et al., 2014; Cappuccio et al., 2010), we hypothesize that improved sleep may be such underlying mechanism.

Therefore, our study aimed to investigate the associations between the amount and timing of objectively assessed MVPA with sleep quality in the general population.

## 2 Material and methods

### 2.1 Study design and study population

**Fig. 1:**
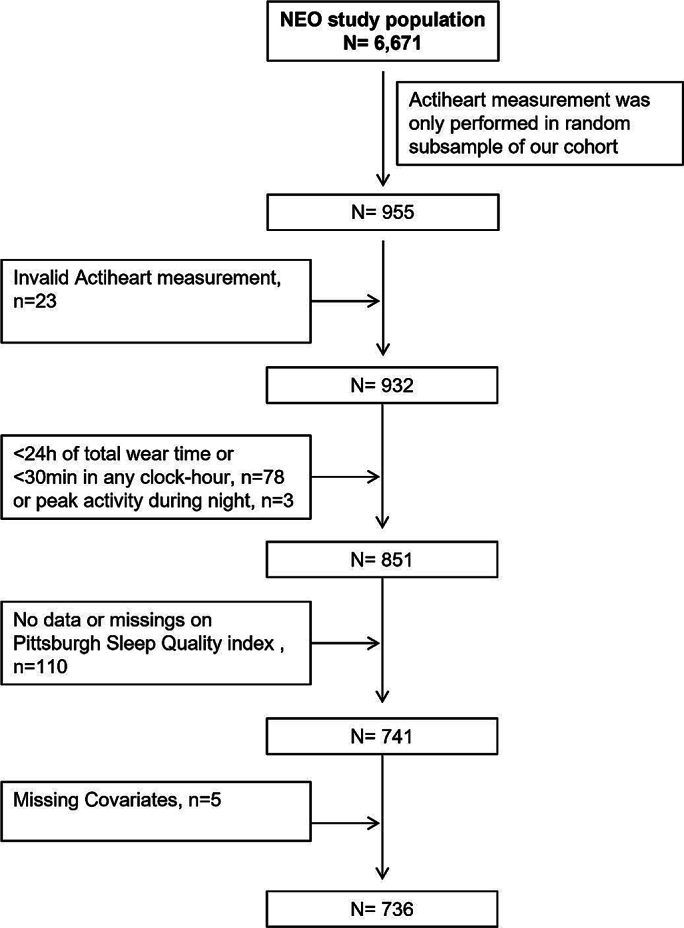
flowchart to illustrate selection of study population. NEO, Netherlands Epidemiology of Obesity study.

This study is a cross-sectional analysis of the baseline measurements of the Netherlands Epidemiology of Obesity (NEO) study. The NEO study is an ongoing population-based prospective cohort study that aims to investigate pathways that lead to obesity-related diseases. In total, 6671 middle-aged men and women have been included in the period between 2008 and 2012, with an oversampling of adults with overweight or obesity. Men and women living in the greater area of Leiden (The Netherlands), aged between 45 and 65 years and with a self-reported BMI of at least 27 kg/m2, were invited to participate in the NEO study. Additionally, all inhabitants between 45 and 65 years from the municipality Leiderdorp were invited for participation irrespective of their BMI, allowing for a reference distribution of BMI. Participants completed questionnaires about demographic, lifestyle and clinical information at home. Participants visited the NEO study center of the Leiden University Medical Center (LUMC) after an overnight fast of at least ten hours for an extensive physical examination including blood sampling and anthropometric measurements. Since the baseline visit, participants are followed for the incidence of obesity-related diseases via postal follow-up questionnaires and through the medical records of their general practitioner. The study design was approved by the Medical Ethical Committee of the LUMC and all participants gave written informed consent. The study design and data collection of the NEO study have been described in detail elsewhere (de Mutsert et al., 2013). In the present analysis, we only included the random subset of participants who carried an accelerometer (*n* = 955) and excluded those with an invalid measurement (*n* = 23), with total wear time < 24 h or with <30 min in any clock-hour (*n* = 78), participants with an activity peak during the night (*n* = 3). Further, we excluded participants that were enrolled in the NEO study before July 2009, as the sleep quality questionnaire was added to the NEO study baseline measurements hereafter. Lastly, we excluded those with missing covariates (*n* = 5) (Fig. 1), resulting in our study sample of *n* = 736.

### 2.2 Physical activity

A random selection of participants of the NEO study population were fitted with a combined uniaxial acceleration and heart rate monitor (Actiheart, CamNtech Ltd., UK) to measure daily levels of activity for four consecutive days. The monitor was placed on the chest, and participants were instructed to wear it continuously and to maintain their habitual level of activity. Details have been described previously (Winters-van Eekelen et al., 2021). Acceleration and heart rate were recorded in 15-s epochs and a branched equation algorithm was used to estimate physical activity energy expenditure (Brage et al., 2004). In addition, a random subgroup of participants (*n* = 132) performed an eight-minute ramped step test to calibrate the individual heart rate response to activity intensity (Brage et al., 2005). For participants without individual calibration, a group calibration was applied that was based on age, sex, and sleeping heart rate (Winters-van Eekelen et al., 2021). Physical activity energy expenditure was used to determine the time spent in different physical activity intensities expressed as metabolic equivalents of task (MET). Moderate-to-vigorous physical activity was defined as an activity intensity >3MET.

From these data, participants were categorized as performing most MVPA in the morning (06:00–12:00), afternoon (12:00–18:00), and evening (18:00–24:00) (van der Velde et al., 2022). A minimum difference of 5 % was used to distribute participants to one of these time windows. If the share of MVPA in each block differed from the others by less than 5 %, then it was classified as an even distribution of activity (van der Velde et al., 2022). For example, an individual with 40 %, 30 % and 30 % of total MVPA in the morning, afternoon, and evening, respectively, was classified as most active in the morning. Whereas an individual with 35 %, 33 %, and 32 % total MVPA in the morning, afternoon, and evening, respectively, was classified as having an even distribution.

### 2.3 Sleep

Sleep quality was assessed with the Pittsburgh Sleep Quality index (PSQI) (Buysse et al., 1989). This 24-item questionnaire generates seven component scores for sleep quality over a one-month period: sleep quality, sleep latency, sleep duration, habitual sleep efficiency, sleep disturbances, use of sleep medications and daytime dysfunction. Each component is scored on a range from 0 to 3, with 3 indicating worst score. The sum of these seven components yields a global score of subjective overall sleep quality (range 0–21). For our analyses, we dichotomized each of the seven components of the PSQI as good (a score of 0 or 1) and poor (a score of 2 or 3). In addition, we dichotomized the global score of overall sleep quality as good (total PSQI score ≤ 5) and poor (total PSQI score > 5). Sleep duration (also one of the components) was used as a continuous outcome in minutes as well. We calculated also from the PSQI, the midpoint of sleep by taking the middle point of (clock) time of the total time that was spent in bed as a measure of chronotype (Kantermann and Burgess, 2017).

In addition, risk of obstructive sleep apnea was assessed with the Berlin questionnaire (Netzer et al., 1999), which consists of ten questions that form three categories i.e. snoring, daytime somnolence, and hypertension and BMI. Individuals were classified as having a high (≥two categories with positive score) or a low (≤1categories with positive score) risk of having obstructive sleep apnea.

### 2.4 Data collection

At the study center, a bio impedance scale (TBF-310, Tanita International Division, UK) was used to estimate body weight and percentage of total body fat, with the participant not wearing shoes and one kg was subtracted from body weight to account for the weight of clothing. By questionnaire, participants reported their date of birth and sex. Highest level of education was reported in ten categories (according to the Dutch education system) and grouped into high (including higher vocational school, university, and post-graduate education) and low education (reference category). Participants reported ethnicity by self-identification, which we grouped into white (reference) or other. Tobacco smoking was divided into three categories: current, former, and never smoking (reference). The Beck Anxiety Inventory was used to estimate the level of anxiety based on 21 multiple choice questions (Beck et al., 1988). Severity of depressive symptoms was estimated with the 28-item Inventory of Depressive Symptomatology (Rush et al., 1986). Habitual alcohol (in g/day) and coffee consumption (in units/day) was estimated using a semi-quantitative food frequency questionnaire (Siebelink et al., 2011; Verkleij-Hagoort et al., 2007).

### 2.5 Statistical analyses

In the NEO study, individuals with a BMI of 27 kg/m2 or higher were intentionally oversampled. To correctly represent baseline associations in the general population, adjustments for the oversampling of individuals with a BMI ≥27 kg/m^2^ were made. This was done by weighting all participants toward the BMI distribution of participants from the Leiderdorp municipality, whose BMI distribution was similar to the BMI distribution of the general Dutch population (Ministerie van Volksgezondheid Welzijn en Sport Dutch Ministry of Health Welfare and Sport, 2017). All results were based on weighted analyses. Consequently, the results apply to a population-based study without oversampling of individuals with a BMI ≥27 kg/m2 (Lumley, 2004). Population characteristics were summarized as mean [standard deviation (SD)], median (25th, 75th percentiles) or as percentage for the total population and stratified by timing of MVPA. As a consequence of the weighted analyses, no absolute numbers could be given, only proportions.

Using logistic regression models, we examined associations between the total amount of MVPA (in hours per day) and a poor outcome of overall sleep quality (global PSQI score) and each of the seven sleep components (i.e. poor subjective sleep quality, poor sleep latency, poor sleep duration, poor sleep efficiency, sleep disturbances, use of sleep medication, dysfunction during daytime. Associations from these logistic regression models were expressed as OR with 95 % CI. Subsequently, using logistic regression analyses, we examined associations between time-of-day in which most MVPA was performed and a poor outcome of overall sleep quality and each of the seven sleep components, in comparison with an even distribution of MVPA as the reference category. Furthermore, we examined associations between the amount of MVPA and time-of day of MVPA with sleep duration on a continuous scale (minutes) using linear regression analyses and estimated regression coefficients with 95 % CI. Finally, we examined the associations between the amount of MVPA and time-of-day of MVPA and odds of obstructive sleep apnea with logistic regression models. For all analyses, we performed crude analyses and adjusted for potential confounding in 3 models. Associations in model 1 were adjusted for age, sex, and level of education. Associations in model 2 were additionally adjusted for ethnicity, coffee consumption, alcohol intake, smoking, total body fat, anxiety symptoms, and depression symptoms. Associations in model 3 were additionally adjusted for the midpoint of sleep. The associations between time-of-day of MVPA and sleep outcomes in models 2 and 3 were additionally adjusted for the total amount of MVPA per day. We used STATA version 16.1 (StataCorp LP, College Station, TX, USA) for all analyses.

## 3 Results

### 3.1 Characteristics

In total, we included *n* = 736 participants (57 % women), with mean (SD) age of 56 (6) years and BMI 26.1 (4.2) kg/m2. The population characteristics are presented in Table 1, stratified by the time window in which most MVPA was performed. The participants who did not complete the PSQI were more often women (72 %) and were otherwise comparable to the study population included in the analyses.

**Table 1:** Characteristics of participants from the Netherlands Epidemiology of Obesity study (2008–2012)*, stratified by the time of day when most moderate-to-vigorous physical activity was performed.

	Total population	Even distribution of MVPA	Most MVPA in morning	Most MVPA in afternoon	Most MVPA in evening
Proportion of population		12 %	20 %	61 %	7 %
Sex (% women)	57	53	55	57	59
Age (years)	56 (6)	56 (6)	55 (6)	56 (6)	55 (7)
Education (% high)	42	44	40	40	57
Ethnicity (% whites)	96	95	92	96	100
BMI (kg/m^2^)	26.1 (4.2)	26.2 (3.8)	25.9 (4.1)	26.1 (4.4)	26.5 (4.0)

Total body fat (%) Men Women					
Smoking (% current)	16	16	16	15	20
MVPA (hour/day)	1.5 (1.1)	1.9 (1.7)	1.7 (1.0)	1.4 (1.0)	1.1 (0.6)
Midpoint of sleep (clock time in hour)	3.2 (0.9)	3.1 (0.8)	2.9 (0.7)	3.2 (0.8)	3.8 (1.4)

Poor overall sleep quality (%)^a^	30	28	30	30	36
Sleep duration (hours)	7.0 (1.1)	6.9 (0.9)	6.9 (1.0)	7.0 (1.1)	7.2 (1.3)
Risk of obstructive sleep apnea (% high risk)	18	16	18	19	15

*Limited to those with objectively assessed physical activity data and a complete Pittsburgh sleep quality index questionnaire. Results are based on analyses weighted toward the BMI distribution of the general population (n = 736) and presented as mean (SD) or percentages. MVPA, moderate-to-vigorous physical activity; BMI, body mass index. ^a^ Poor overall sleep quality was defined as a total score > 5 on the Pittsburgh sleep quality index questionnaire.

Participants who performed most MVPA in the evening had a later midpoint of sleep clock time: 3.8 (1.4) hours than participants with most MVPA in the morning (clock time: 2.9 (0.7) hours) or afternoon (clock time: 3.2 (0.8) hours). Participants who performed most MVPA in the evening most frequently reported poor overall sleep quality (36 % of participants) while this was least reported by participants that evenly spread their MVPA over the day (28 %).

### 3.2 Amount of MVPA and sleep quality

Associations between total amount of daily MVPA and sleep characteristics are presented in Table 2. There were no apparent association between the total amount of MVPA and overall sleep quality score, nor with most of the individual components of the PSQI, although confidence intervals were wide. From the individual sleep components, one hour of MVPA per day was associated with lower odds of fatigue-related dysfunction during daytime (OR [95 % CI]: 0.54 [0.32–0.93] in the fully adjusted model). Further, after full adjustment, there were no associations between amount of MVPA and sleep duration (in minutes); B: −1.49 [−6.59–3.62]) or being at risk of obstructive sleep apnea; OR:0.90 [0.71–1.15] per hour MVPA per day.

**Table 2:** Associations between the amount of moderate-to-vigorous physical activity and total overall sleep quality score and each of the seven sleep components in participants from the Netherlands Epidemiology of Obesity study (2008–2012)⁎.

		Poor overall sleep quality	Poor subjective sleep quality	Poor sleep latency	Poor sleep duration	Poor sleep efficiency	Sleep disturbances	Sleep medication	Dysfunction during daytime
		*OR (95 % CI)*	*OR (95 % CI)*	*OR (95 % CI)*	*OR (95 % CI)*	*OR (95 % CI)*	*OR (95 % CI)*	*OR (95 % CI)*	*OR (95 % CI)*
Amount of MVPA (hours /day)	Model 1	0.84 (0.67–1.05)	0.94 (0.71–1.25)	1.02 (0.81–1.27)	1.11 (0.83–1.48)	0.86 (0.59–1.24)	0.99 (0.68–1.45)	1.11 (0.77–1.59)	0.47 (0.30–0.75)
	Model 2	0.90 (0.72–1.13)	1.06 (0.81–1.38)	1.07 (0.86–1.33)	1.23 (0.96–1.59)	0.89 (0.62–1.29)	1.13 (0.83–1.54)	1.25 (0.93–1.69)	0.55 (0.33–0.93)
	Model 3	0.92 (0.73–1.15)	1.05 (0.80–1.38)	1.09 (0.87–1.36)	1.20 (0.92–1.56)	0.89 (0.61–1.30)	1.16 (0.85–1.58)	1.22 (0.90–1.67)	0.54 (0.32–0.93)

Model 1 associations were adjusted for age, sex, and educational background.

Model 2 associations were additionally adjusted for ethnicity, coffee- and alcohol consumption, smoking, total body fat, and symptoms of depression and anxiety.

Model 3 associations were additionally adjusted for the midpoint of sleep.

MVPA, moderate-to-vigorous physical activity.

⁎ Limited to those with objectively assessed physical activity data and a complete Pittsburgh sleep quality index questionnaire. Results are based on logistic regression analyses weighted toward the BMI distribution of the general population (*n* = 736) and presented as OR and 95 % CI.

### 3.3 Timing of MVPA and sleep quality

**Fig. 2:**
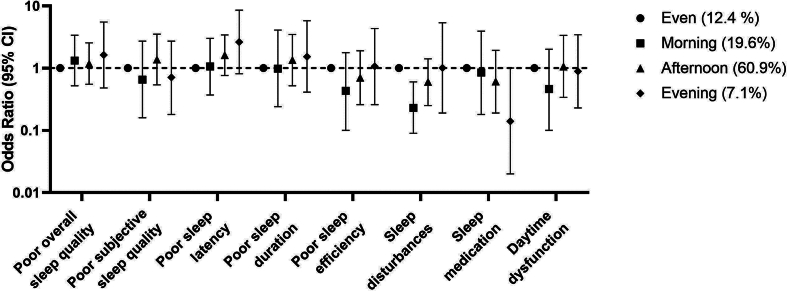
Associations between timing of moderate-to-vigorous physical activity and a poor outcome of overall sleep quality and poor outcome on the seven components of the Pittsburgh sleep quality index questionnaire, compared with an even distribution of moderate-to-vigorous physical activity throughout the day in participants from the Netherlands Epidemiology of Obesity study (2008–2012)*. *Limited to those with objectively assessed physical activity data and a complete Pittsburgh sleep quality index questionnaire. Results were based on logistic regression analyses weighted toward the BMI distribution of the general population (n = 736) and presented as OR with 95 % CI. Associations were adjusted for age, sex, education level, coffee intake, alcohol intake, smoking, total daily amount of moderate-to-vigorous physical activity, total body fat, level of anxiety, level of depression, and midpoint of sleep.

Fig. 2 visualizes the associations between timing of MVPA with overall sleep quality and all individual sleep components (model 3). There were no associations between time-of-day of MVPA and most sleep components. However, participants who performed most MVPA in the morning had lower odds of sleep disturbances than participants who evenly spread MVPA over the day (OR [95 % CI]: 0.23 [0.09–0.60]). Timing of MVPA was not associated with sleep duration (in minutes): performing MVPA in the morning (regression coefficient in minutes [95 %] CI: 7.6 [−12.8–27.9]), afternoon (5.5 [−9.7–20.7]) and evening (9.1 [−17.4–35.6]) as compared with participants with an even distribution of MVPA over the day. Further, compared with an even distribution of MVPA, the ORs (95 % CI) of being at risk of obstructive sleep apnea were 0.91 [0.38–2.15) for performing MVPA in the morning, 1.20 [0.64–2.25] in the afternoon, and 0.78 [0.27–2.30] in the evening.

## 4 Discussion

Our study aimed to examine associations between the amount and the timing of MVPA with sleep quality in the general population. Neither the amount nor the timing of MVPA was associated with sleep quality, sleep latency, duration of sleep, sleep efficiency and sleep medication use, and confidence intervals were large. A larger amount of MVPA was associated with lower odds of fatigue-related dysfunction during daytime and performing most MVPA in the morning was associated with reduced odds of sleep disturbances compared with evenly distributing MVPA over the day.

While the wide confidence intervals around our estimates, in particular those concerning the results of timing of MVPA, suggest that our sample size is too small to obtain robust findings, our results do not seem to support results from previous randomized controlled trials demonstrating that performing physical activity regularly (in total ≥ 1 weeks with exercise) exerts moderate-to-large beneficial effects on sleep quality (Kredlow et al., 2015). These previous studies investigated the effects of exercise in controlled human intervention studies (Jurado-Fasoli et al., 2020; Reid et al., 2010; Jiménez-García et al., 2021). Exercise is a component of MVPA that is structured and performed for consecutive time periods (Caspersen et al., 1985). As such, it is possible that the beneficial effects of MVPA on sleep only occur if the activity is performed at higher intensity and maintained for a longer period or on a regular basis. Studies that investigated the association between objectively measured MVPA and subjective sleep characteristics in the general population are scarce. A recent study in 96,476 UK Biobank participants indicated that performing more MVPA per day (measured with wrist-worn accelerometers) was associated with a lower risk of all-cause mortality (Strain et al., 2020). Since poor sleep has been related to poor health outcomes (Javaheri et al., 2011; Rutters et al., 2016; Xi et al., 2014; Cappuccio et al., 2010; Morssinkhof et al., 2020), it may be possible that there actually is an association between the amount of MVPA and sleep characteristics, but that our sample size was too small to detect this.

Nonetheless, in our study, we did observe that the amount of MVPA per day was related with lower odds of fatigue-related dysfunction during daytime. The PSQI measures dysfunction during daytime with questions about the difficulty to remain awake when doing daytime activities, and the enthusiasm to execute these activities. A possible explanation for the association between MVPA and less daytime dysfunction may be that people who perform more MVPA also have a better mood, as reported previously (Carriedo et al., 2020; Hajo et al., 2020), and are therefore more energetic during the day. In our analyses, we adjusted for the potential confounding effects of depressive symptoms on the associations between MVPA and sleep. The addition of this variable did, however, not largely change our results, which suggests that mood only minimally influenced the associations.

Additionally, we hypothesized that performing most MVPA in the afternoon and evening would be associated with optimal sleep quality. This hypothesis was based on previous studies that showed that MPVA in the afternoon and evening was associated with most optimal metabolic benefits (Mancilla et al., 2021; Savikj et al., 2019; van der Velde et al., 2022), and that high-quality sleep is important for metabolic health (Bacaro et al., 2020; Javaheri et al., 2011; Rutters et al., 2016; Xi et al., 2014; Cappuccio et al., 2010). However, performing most MVPA in the afternoon or evening was not associated with improved sleep characteristics, except for sleep disturbances, and could therefore not explain the previous findings. If anything, the ORs of poor sleep outcome were even lower in the group with most MVPA in the morning than in the afternoon or evening (Fig. 2). To date, most previous studies on the association between time-of-day of physical activity and sleep were controlled experiments that examined if evening exercise conferred detrimental effects on sleep quality. A meta-analyses on 23 controlled intervention studies that compared an acute bout of evening exercise to a no-exercise control in healthy adults did not show detrimental effects of evening exercise on sleep quality (measured using e.g. actigraphy, accelerometery, and polysomnography), except when vigorous exercise ended ≤1 h before bedtime (Stutz et al., 2019). In a previous observational study in 129 adolescents and using an activity monitor to measure both MVPA and sleep, evening MVPA was associated with longer sleep onset latency and higher wake after sleep onset, although this association was only present in girls (Jurić et al., 2022). On the other hand, low intensity stepping exercises during the evening showed a greater improvement on sleep quality than exercises in the morning in older adults (Seol et al., 2021). In our study, performing most MVPA in the morning was associated with a lower odds of sleep disturbances as compared with distributing MVPA evenly over the day. Performing most MVPA in the morning may reflect a general preference for early activity and sleep, i.e. an early chronotype, and this chronotype is generally associated with better health outcomes, compared to a late chronotype (Merikanto et al., 2013; Wong et al., 2015). Additional adjustment for midpoint of sleep as a proxy for chronotype in our models, did not impact our findings. The lack of an association between MVPA in the afternoon and evening with improved sleep characteristics suggests that the association between MVPA in the afternoon and evening with improved metabolic outcomes that was found in previous studies (Mancilla et al., 2021; Savikj et al., 2019; van der Velde et al., 2022) is likely not via improved sleep quality, although differences in activity protocols may affect such effects. This finding is in line with previous studies that also observed that the relation between physical activity during the night (as with night-shift work) and adverse metabolic health, was independent of poor sleep (Lim et al., 2018; Leproult et al., 2014). Combined, these results may favor that the association between the timing of physical activity and metabolic health goes via other mechanisms than improved sleep.

Strengths of this study include the objective measurement of MVPA over four days using the ActiHeart measurement and the use of a validated questionnaire to assess multiple components of sleep quality, (van der Velde et al., 2022). With the ActiHeart, heart rate and acceleration are also measured in 15-s epochs. Therefore, this method gives a more accurate reflection of the intensity of physical activity as compared to accelerometers that do not measure heart rate. Importantly, the high frequency of measurement epochs also gives a precise estimation of the time-of-day at which the MVPA occurred. A weakness of this study was that participants wore the ActiHeart only for four days, which yielded limited data on the day-to-day variation in physical activity and most specifically the variation in physical activity during week and weekend days. Consequently, misclassification of timing of MVPA may have occurred by summarizing data into an average 24-h period. Another weakness of this study is the lack of additional objective measurements to assess sleep quality, such as polysomnography. Since polysomnography is a laborious measurement method, it is unfortunately unsuitable to use in large-scale observational studies. Furthermore, the cross-sectional design precludes from any causal inferences. Subsequently, reverse causation may be present. For instance, it is plausible that individuals who function poorly during daytime are also less inclined to engage in MVPA. Residual confounding may still be present as well. For example, no information was available regarding the context of the activities that were performed, specifically whether the activities were carried out indoors or outdoors. Since sleep is tightly connected to the circadian rhythm, which is most sensitive to (day)light exposure (Morris et al., 2012), it could be argued that MVPA performed outdoors is more strongly associated with good sleep quality compared with performing MVPA indoors. Lastly, the data was collected between 2008 and 2012 and this may be considered not current. However, (self-reported) health behaviors as physical activity (the proportion of adults meeting physical activity guidelines) (Statistics Netherlands (CBS) And National Institute for Public Health and the Environment (RIVM), 2023), and sleep duration (Tiemeier and van Someren, 2017) have not changed in the Dutch general population in the past 15 years. Therefore, we expect that our findings will not differ from a study that would have collected data more recently.

In conclusion, in our cross-sectional analysis the amount of MVPA was associated with functioning during daytime, whereas performing most MVPA in the morning associated with fewer sleep disturbances. However, there were no apparent associations of timing of MVPA with overall sleep quality nor the other components. Thus, it is unlikely that improved sleep quality underlies the previously observed associations between performing most MVPA in the afternoon or evening and metabolic health. However, given the wide confidence intervals in our study, larger studies are needed that scrutinize the relation between timing of MVPA and sleep.

## CRediT authorship contribution statement

**Charlotte Andriessen:** Writing – original draft, Visualization, Formal analysis. **Femke Rutters:** Writing – review & editing, Funding acquisition. **Joris Hoeks:** Writing – review & editing, Supervision, Funding acquisition. **Andries Kalsbeek:** Writing – review & editing. **Raymond Noordam:** Writing – review & editing, Resources. **Frits R. Rosendaal:** Writing – review & editing, Resources, Project administration. **Diana van Heemst:** Writing – review & editing, Resources. **Jean-Pierre Després:** Writing – review & editing, Funding acquisition. **Parminder Raina:** Writing – review & editing, Funding acquisition. **David J.T. Campbell:** Writing – review & editing, Funding acquisition. **Patrick Schrauwen:** Writing – review & editing, Supervision, Funding acquisition. **Renée de Mutsert:** Writing – review & editing, Project administration, Funding acquisition. **Jeroen H.P.M. van der Velde:** Writing – review & editing, Visualization, Formal analysis.

## Funding

This study was funded by The Netherlands Organization for Health Research and Development (ZonMw) [459001021], Dutch Diabetes Research Foundation (Diabetes Fonds) [2019.11.101], the Canadian Institutes of Health Research (CIHR) [TNC-174963], and Health-Holland [LSHM20107]. This collaborative project is co-financed with PPP-allowance made available by Health-Holland, Topsector Life Sciences & Health, to stimulate public-private partnerships.

P.R. holds the Raymond and Margaret Labarge Chair in Optimal Aging and Knowledge Application for Optimal Aging and Tier 1 Canada Research Chair in Geroscience.

## Declaration of competing interest

The authors declare that they have no known competing financial interests or personal relationships that could have appeared to influence the work reported in this paper.

## Data Availability

Due to the privacy of the participants of the NEO study and legal reasons, we cannot publicly deposit the data. In addition, NEO study participants did not sign informed consent to make their data publicly available. Data will be made available upon reasonable request to qualified researchers according to the NEO study research procedure. Data requests should be sent to the NEO Executive Board, who can be contacted via https://www.lumc.nl/org/neo-studie/contact/.
